# Leaf allocation improves predictability of interspecific growth rates in a broadleaf deciduous temperate forest

**DOI:** 10.1002/ecy.70203

**Published:** 2025-09-13

**Authors:** Minh Chau N. Ho, Michael Kalyuzhny, María Natalia Umaña, Annette M. Ostling

**Affiliations:** ^1^ Department of Ecology and Evolutionary Biology University of Michigan Ann Arbor Michigan USA; ^2^ Department of Integrative Biology University of Texas at Austin Austin Texas USA; ^3^ Present address: Department of Ecology, Evolution and Behavior, The Alexander Silberman Institute of Life Sciences Hebrew University of Jerusalem Jerusalem Israel

**Keywords:** functional traits, leaf allocation, leaf area index, leaf mass per area, relative growth rates, temperate deciduous forest, trait–performance relationships

## Abstract

Understanding the relationships between species' demography and functional traits is crucial for gaining a mechanistic understanding of community dynamics. While leaf morphology represents a key functional dimension for plants worldwide (i.e., the leaf economics spectrum), its ability to explain variation in trees' life history strategies remains limited. Plant growth is influenced by both leaf morphology and allocation; hence, incorporating both dimensions is essential but rarely done. Additionally, trait–performance relationships have mainly been studied in tropical communities, leaving gaps in our understanding of temperate forests where different seasonality patterns may alter these relationships. We examined whether species' leaf area index (leaf area per crown size, LAI), a measure of leaf allocation, explains the variation of juvenile tree species' potential growth rates in a winter‐deciduous broadleaf forest. LAI has not been characterized as a species‐level trait, but its ability to predict plant productivity at the ecosystem scale highlights its potential for explaining plant growth. We evaluated species' maximum LAI both individually and in conjunction with wood density (WD) and leaf mass per area (LMA). We expected that models would improve when both leaf morphology (LMA) and leaf allocation (LAI) were included and that species with denser crowns would have higher potential growth rates. LAI and LMA were significant predictors of growth but only when both were incorporated, and together explained a high proportion of species' growth variations (*R* ^2^_adj_ = 0.59). We found evidence of a trade‐off between LAI and LMA, with a negative relationship between them and each having a positive influence on species' growth, suggesting that there are multiple allocation strategies to achieve fast growth. A surprisingly positive LMA–growth relationship contrasts with observations from tropical forests. We did not find significant relationships with WD in this forest. Our results highlight that incorporating leaf allocation improves models of trait–performance relationships. They also suggest, in agreement with the limited literature, that temperate forests may exhibit different trait–performance relationships from those of tropical forests, where LMA is negatively related to growth and WD is often important. Clarifying the details and contexts of trait–performance relationships is crucial for applying the functional trait framework to understanding community structure and dynamics of forests globally.

## INTRODUCTION

Organisms' phenotypes (i.e., a combination of multiple traits) mediate how species interact with each other and the environment, ultimately affecting their performance (Violle et al., 2007). The use of such phenotypic traits to predict species' performance has been embraced by ecologists as a tool for studying communities where long‐term performance data are not available. These trait–performance relationships also provide a deeper biological understanding of species' life history strategies, such as the growth–mortality trade‐off (Wright et al., 2010), and community dynamics such as coexistence through successional niches (Adler et al., 2013).

However, plant traits are limited in their ability to explain interspecific growth variations (Paine et al., 2015; Visser et al., 2016). In trees, wood density (WD) has emerged as a strong correlate of growth, with species having harder/denser woods exhibiting slower growth rates (Francis et al., 2017; King et al., 2006). However, the coefficients of determination are often low (between 0.10 and 0.30). Leaf mass per area (LMA), a key component of plants' leaf economics spectrum (Wright et al., 2004), explains less interspecific variation than WD (Visser et al., 2016). Fast‐growing species tend to have lower LMA, which can generate thinner, broader leaves better at intercepting light for photosynthesis (Martínez‐Vilalta et al., 2010; Reich et al., 1992). One potential explanation for LMA's limited effect is that this trait represents the morphology of individual leaves and fails to capture the whole plant investment in photosynthetic tissues (Yang et al., 2018). Integrating traits that quantify allocation to leaves in the crown should enhance our ability to explain tree species' growth differences.

Prior work from the plant ecophysiology literature theoretically recognizes plants' relative growth rates (RGRs) as a product of leaf morphology (e.g., LMA) and leaf allocation (e.g., leaf weight ratio) and has amassed empirical support for their combined influence in studies within species or among small groups of species (Poorter, 1989). RGR is defined as a change in biomass, diameter, or height relative to the initial value over time. Measures of leaf morphology such as LMA fit within the leaf economics spectrum framework, but measures of plants' leaf allocation have mostly been ignored in the trait‐based community ecology literature (Yang et al., 2018, but see Umaña et al., 2021). Furthermore, plants can maximize their growth rates through different combinations of leaf allocation and leaf morphology (Bonser, 2006). That is, plants can achieve the same level of productivity by investing in more productive leaves or in more leaves overall, but this latter dimension has received relatively little attention in the functional trait literature.

Mechanistic models of trait–performance relationships suggest that trees' leaf area index (LAI), a measure of leaf allocation, has a large and positive influence on species' carbon assimilation rates (Sterck et al., 2011). LAI is the total leaf area (TLA) per crown projection area (CPA), and most prior work has focused on the positive relationship between LAI and primary productivity at large spatial scales (le Maire et al., 2019; Reich, 2012). Not much is known about interspecific LAI variability, but its positive correlation with plant productivity suggests an important influence on plant growth. Prior studies have used different measures of leaf allocation with mixed results (Iida et al., 2014; Rubio et al., 2021; Takahashi et al., 2001; Umaña et al., 2021), and almost no studies have incorporated measures of leaf allocation with leaf morphology to explain species' growth rates (but see Rubio et al., 2021; Umaña et al., 2021). We propose studying LAI in the context of life history strategies and incorporated with leaf morphology. Early‐successional species often scatter their leaves in multiple layers, which may lead to higher LAI, while late‐successional species arrange their leaves in monolayers (Givnish, 2020). LAI is expected to vary widely within species, and we propose that the highest levels of LAI achieved by a species may provide a useful species‐level measure of leaf allocation.

Here, we evaluate whether LAI provides good predictive power of growth rates both on its own and as part of a model that incorporates LMA (a key measure of leaf morphology) and WD (another important dimension of plant growth). We focus our study on saplings growing in the light‐limited forest understory where traits influencing light harvesting are important, and because juveniles are nonreproductive and net carbon gain can be allocated more directly to growth. We examine these trait–performance relationships across dominant tree species in a temperate broadleaf forest in Michigan, where winter deciduousness results in synchronous annual leaf loss for all species (Runkle, 1989). In tree communities, trait–performance relationships have been examined more extensively in the tropics than in temperate forests (Martínez‐Vilalta et al., 2010), but seasonal dynamics in temperate forests may alter some of these relationships (Janse‐Ten Klooster et al., 2007). Here, we add to the literature on temperate forests to broaden our understanding of trait–performance relationships.

We use these traits to ask two questions. First, does considering both leaf allocation and leaf morphology improve our models of species' maximum potential growth rates? We hypothesize that both leaf allocation and leaf morphology are important components of growth. We expect that models will significantly improve when both dimensions are incorporated, with higher coefficients of determination and better predictions of growth. Second, how do leaf and wood traits relate to species' growth rates in a temperate forest? Based on the literature reviewed above, we hypothesize that at the sapling stage, species with denser crowns (higher LAI values), more acquisitive leaves (lower LMA values), and lower WDs will be better able to harvest light for carbon gain and have lower carbon construction costs, generating higher growth rates. As these expectations were extrapolated from tropical forest studies, we expect that some may need revision due to the influence of different seasonalities in our temperate forest (Janse‐Ten Klooster et al., 2007).

## MATERIALS AND METHODS

### Study site

We conducted this study in a temperate forest at the Edwin S. George Reserve (Livingston County, 42°27′46.5″ N, 84°00′21.9″ W), a 525‐ha ecological reserve in southeast Lower Michigan. The site receives an average annual precipitation of 857 mm and temperatures range from −9.7 to 0.3°C in January to 15.0 to 27.4°C in July over the last 30‐year period (NOAA, 2023). In the early 18th century, the area was used as cultivated fields, woodlots, and pasturage, then as a game preserve until 1930 (Cantrall, 1943). The reserve is fully fenced and contains a population of white‐tailed deer (*Odocoileus virginianus*). The reserve has a history of managed burning by Indigenous Nations, but subsequent fire suppression by European colonizers has resulted in forest mesophication from oak‐hickory to a more mesic community of black cherries and red maples (Allen et al., 2020; Cantrall, 1943).

In 2003, a 12‐ha forest dynamic plot (Big Woods Plot) was established where all woody stems ≥3.2 cm in dbh were identified, tagged, and measured for dbh (Allen et al., 2020). Subsequent censuses in 2008–2010 and in 2014 remeasured dbh for all stems and integrated all new recruits following protocols established by the Smithsonian Forest Global Earth Observatory (ForestGEO). In 2008–2010, the plot was expanded another 11 ha to form the current plot size of 23 ha. In 2014, the plot contained 30 tree species belonging to 14 families, with 24,912 living stems and 6,920,043cm^2^ of the basal area.

In our study, we focused on the 11 most abundant tree species that were identifiable at the sapling stage. These species represented 86.6% of living tree stems and 48.5% of living tree basal area in 2014 (Table 1). Some species with high stem densities were excluded because they were difficult to identify or find at the sapling stage: *Carya glabra* (1176 stems), *Quercus velutina* (962 stems), *Quercus ×hawkinsiae* (557 stems), *Carya ovata* (184 stems), *Quercus rubra* (159 stems), and *Quercus ×palaeolithicola* (158 stems).

**TABLE 1:** Total basal area (BA, in square centimeters, calculated as $\pi r^{2}$ from dbh) and stem abundance of the selected deciduous temperate tree species in the Michigan Big Woods.

Family	Latin name	Authority	Common name	BA	Stems	dbh_sap_
Rosaceae	*Prunus serotina*	Ehrhart	Black cherry	1,019,090	8548	7.5
Sapindaceae	*Acer rubrum*	Linnaeus	Red maple	699,292	6904	5.0
Rosaceae	*Amelanchier arborea*	(F. Michx.) Fernald	Serviceberry	49,883	2839	3.5
Fagaceae	*Quercus alba*	Linnaeus	White oak	1,371,052	1167	7.5
Ulmaceae	*Ulmus americana*	Linnaeus	American elm	52,289	658	5.0
Lauraceae	*Sassafras albidum*	(Nuttall) Nees	Sassafras	107,897	557	5.0
Cornaceae	*Cornus florida*	Linnaeus	Flowering dogwood	10,560	384	3.5
Betulaceae	*Ostrya virginiana*	(Miller) K. Koch	Hophornbeam	17,353	291	7.5
Tiliaceae	*Tilia americana*	Linnaeus	American basswood	18,197	115	7.5
Fagaceae	*Fagus grandifolia*	Ehrhart	American beech	10,716	70	7.5
Oleaceae	*Fraxinus americana*	Linnaeus	White ash	728	27	7.5

*Note*: dbh_sap_ (in centimeters) indicates the dbh cutoff that defines a sapling, based on literature values or congeners (see *Materials and methods* and Appendix S1: Section S1).

### Growth rates

For each species, we calculated RGRs for saplings (RGR_sap_, in centimeters per centimeter per year) as $\mathrm{RG}R_{\mathrm{sap}}=\ln\left(\frac{{\mathrm{dbh}}_{f}}{{\mathrm{dbh}}_{i}}\right)/\left(t_{f}-t_{i}\right)$ for initial and final time points, i and f, respectively, pooled across the 2003–2008 and 2008–2014 census intervals. Census data for the Big Woods Plot are publicly available (Allen et al., 2019). We defined saplings as individuals with dbh_*i*_ at or below a species‐specific cutoff informed by the literature. In general, understory species had a cutoff of 3.5 cm dbh, mid‐sized species at 5.0 cm, and taller species at 7.5 cm (Appendix S1: Section S1). We omitted individuals growing at the plot's edges or along major roads to avoid edge effects (Weemstra et al., 2021). For multistem individuals, we calculated growth rates using only the main stem as the census only began tracking secondary stems in 2014. We omitted individuals with broken and resprouted stems as these may generate negative growth rates that do not reflect biological growth rates of the plant. Aside from removing resprouts, we kept the remaining negative growth rates, as these may have resulted from stem shrinkage and/or measurement error and likely occurred on plants that are alive but growing very little. We calculated species' potential growth rates RGR_sap95_ (in centimeters per centimeter per year) as the 95th percentile of RGR_sap_ for each species (Wright et al., 2010) using the “quantile” function in the R Statistical Software with the default algorithm #7 for a continuous sample quantile type (Hyndman & Fan, 1996; R Core Team, 2023).

### Trait measurements

We measured LAI (in square centimeters per square centimeter) on 23–37 saplings per species as our measure of leaf allocation. Field measurements were collected from mid‐June through mid‐September of 2020 and 2021. We could not control for environmental variation and sample LAI in high light conditions as there are no obvious gaps and few roads throughout the plot (Appendix S1: Figure S3). Instead, healthy, single‐stemmed, free‐standing individuals ≤300 cm tall were sampled widely throughout the plot, focusing on areas with higher juvenile densities for each species to be representative of each species' distribution. *Acer rubrum* was measured up to 350 cm tall, as we were unable to find individuals below 200 cm. Across the duration of the census data, the growing seasons' (defined as May through August) maximum temperatures averaged 24–28°C, minimum temperatures averaged 12–15°C, and total precipitation ranged from 182 to 613 mm. During the two growing seasons when LAI data were collected (2020–2021), averaged maximum temperatures was 26°C for both years, averaged minimum temperatures 14°C for both years, and total precipitation ranged 389–461 mm (NOAA, 2025).

For each sapling, we measured two perpendicular crown diameters including the longest, and calculated CPA as an ellipse ($\mathrm{CPA}=\pi \times \frac{d_{1}}{2}\times \frac{d_{2}}{2}$, in centimeters). We counted all leaves in the crown and collected at least five fully developed and healthy leaves representing their size distribution. We scanned leaves (Epson Perfection V600 Photo) and quantified leaf area using ImageJ, and estimated the average leaf area for each individual (Schneider et al., 2012). We estimated the TLA as the average leaf area times number of leaves, and calculated LAI as TLA/CPA. Additionally, we measured plant height (in centimeters) and stem basal diameter (*d*_b_, in centimeters) to represent plant size, as most individuals were not tall enough to have a dbh measurement. We opted not to use allometric scaling to standardize each species' LAI value because we found no relationship between LAI and plant height or *d*_b_ for most of our species (Appendix S1: Figures S4 and S5). For our analyses, we calculated the 95th percentile of LAI for each species (LAI_95_), using the same method as RGR_sap95_, to represent species' maximum potential LAI. The selection of healthy, leafy individuals and the calculation of LAI_95_ reduced the impact that deer browsing might have on our measure of leaf allocation. Species also varied more in their upper tail of LAI distributions than in their means (Appendix S1: Figure S1).

Leaf morphology was represented by LMA (in kilograms per square meter). To control for intraspecific variation due to environmental conditions, we collected sun‐lit branches from between seven and 13 adult individuals per species growing along major roads using a pole pruner and transported them in black plastic bags. Leaves from only four *Fraxinus americana* trees were collected, as healthy and mature individuals are scarce in the plot. We selected three fully expanded intact leaves with minimal herbivory/pathogens from each individual and scanned each leaf's area using a portable leaf area meter (LI‐3100C; LI‐COR), then oven‐dried leaves for 72 h at 70°C. We calculated species' LMA as averaged dried leaf mass per fresh leaf area. LMA for most species was collected in 2019 (Weemstra et al., 2021); *Amelanchier arborea* and *Cornus florida* were collected in 2021. Leaf morphology and allocation data are available at the University of Michigan's Deep Blue Data (Ho et al., 2025).

WD data came from the Global Wood Dataset using values from North America (BIOMASS package; Chave et al., 2009; Réjou‐Méchain et al., 2017; Zanne et al., 2009).

### Statistical analyses

We explored the effect of incorporating both dimensions of leaf morphology and leaf allocation by testing a model containing these two dimensions against a null model of tree growth. Our null model contained only LMA and WD (Equation 1a), two traits previously identified in the literature to be important for tree growth strategies. Species with more acquisitive leaves (low LMA) and lower construction costs (low WD) tend to have faster growth rates (Wright et al., 2010). We then built an alternative model that added LAI_95_ (Equation 1b) and compared it against the null model. We also compared our alternative model against models containing only LMA or LAI_95_.

(1a) $$H_{0}:\mathrm{RG}R_{\mathrm{sap}95,i}=\alpha_{0}+\alpha_{1}\mathrm{LM}A_{i}+\alpha_{2}WD_{i}+\epsilon_{1,i.}$$

(1b) $$H_{a}:\mathrm{RG}R_{\mathrm{sap}95,i}=\beta_{0}+\beta_{1}\mathrm{LM}A_{i}+\beta_{2}WD_{i}+\beta_{3}\mathrm{LA}I_{95,i}+\epsilon_{2,i.}$$

We evaluated our prediction that the inclusion of species‐level LAI_95_ improves our models of RGR_sap95_ using the *F*‐test (“anova” function in the R Statistical Software) and the Akaike information criterion for small sample sizes (AIC_c_, “model.sel” from MuMIn package; Bartoń, 2024) to compare null versus alternative models (Aho et al., 2014; Johnson & Omland, 2004). We considered the predictive strength of our models using leave‐one‐out cross‐validation with the jackknife method and calculated root mean square error (RMSE, “train” from caret package with method = “LOOCV”; Kuhn et al., 2023). To examine any potentially problematic multicollinearity between traits, we evaluated the variance inflation factor (VIF) for each model using the car package (Fox et al., 2023). Values of VIF≥5 are considered problematic and could lead to erroneous relationships. To understand the influence of individual traits on growth, we examined the values and the significance of the estimated coefficients for each trait within our best model of tree growth.

Species at our study site differed widely in stem abundance (Table 1). To account for this, we ran our linear regression models using weighted least squares (WLS). In WLS, weights can be chosen based on the predicted variance, prior knowledge, or information from a theoretical model (Montgomery et al., 2012). Because the variance in the estimate of a quantile (in our case, RGR_sap95_) is inversely dependent on sample size (Cheung & Lee, 2005), we set the weights for each species as the stem density used to calculate their RGR_sap95_. That is, we gave higher weight to species with larger sample sizes. We also used weighted means when calculating RMSE. While WLS models were the best choice for our dataset, we also ran ordinary least squares (OLS) linear regression (Appendix S1: Section S3).

We fitted all models using the “lm” function in R Statistical Software (version 4.4.1, R Core Team, 2023). LAI_95_ was log_10_‐transformed to attain normally distributed residuals. We standardized all traits by subtracting the mean and dividing by the SD for all observations ($x^{\prime }=\left(x-\bar{x}\right)/\mathrm{SD}\left(x\right)$) to compare the magnitude of their slopes. We kept our response variable in its original units (RGR_sap95_, in centimeters per centimeter per year).

## RESULTS

The alternative model of $\mathrm{LMA}+\mathrm{WD}+\mathrm{LA}I_{95}$ (Equation 1b, *p* = 0.040, $F_{\text{stat}}=\frac{0.035}{0.0073}$ with 3 and 7 df, *R* ^2^_adj_ = 0.53) performed significantly better than the null (Equation 1a, $F_{\text{stat}}=\frac{0.022}{0.014}$ with 2 and 8 df, *R* ^2^_adj_ = 0.095; *F*‐test *p* = 0.022; Table 2), indicating the inclusion of leaf allocation (LAI_95_) along with leaf morphology (LMA) is better at explaining species' variation in growth.

**TABLE 2:** Weighted least squares models of species' potential growth rates (RGR_sap95_).

Model		*p*	*R* ^2^_adj_	*F*‐test	AIC_c_	RMSE_w_	VIF	Int.	Predictor estimates (95% CI)		
									LMA	WD	LAI_95_
1a	LMA + WD	0.28	0.095		−58.0	0.0066	1.29	0.051	0.0058^a^ (−0.0019, 0.013)	0.0031^b^ (−0.0056, 0.012)	
1b	LMA + WD + LAI_95_	**0.040**	0.53	**0.022**	−59.4	0.0050	1.52–1.72	0.057	0.0089^c^ (0.0027, 0.015)	−0.00022^d^ (−0.0072, 0.0067)	0.0091^e^ (0.0017, 0.016)
1c	LMA + LAI_95_	**0.011**	0.59	0.94	−66.8	0.0042	1.46	0.057	0.0090^f^ (0.0035, 0.014)		0.0090^g^ (0.0028, 0.015)

*Note*: All predictor variables were standardized before analysis, while the response variable (growth rates, RGR_sap95_, in centimeters per centimeter per year) was kept in its original units. Coefficient estimates are partial regression coefficient estimates of each variable, in units of RGR_sap95_ which ranged between 0.044 and 0.075 cm cm^−1^ year^−1^.

Abbreviations: AIC_c_, Akaike information criterion for small sample sizes; *F*‐test, model 1a versus 1b, and model 1b versus 1c, *p* values; Int., model intercept; LAI_95_ (in square centimeters per square centimeter), 95th percentile of sapling leaf area index; LMA (in kilograms per square meter), leaf mass per area; *p*, *p* values, with bolded values significant at α=0.05; *R* ^2^_adj_, adjusted coefficient of determination; RMSE_w_, weighted root mean square error from leave‐one‐out cross‐validation analysis; VIF, variance inflation factor of covariates, values ≥5 are considered problematic; WD (in grams per cubic centimeter), wood density.

^a^ *p* = 0.12.

^b^ *p* = 0.43.

^c^ *p* = **0.011**.

^d^ *p* = 0.94.

^e^ *p* = **0.022**.

^f^ *p* = **0.0054**.

^g^ *p* = **0.010**.

**FIGURE 1:**
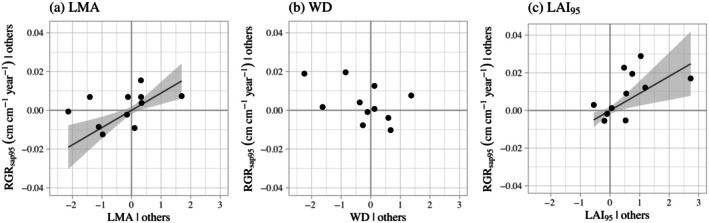
Relationships between individual traits on species' potential growth rates (RGR_sap95_), shown using partial regression plots of our alternative model to include wood density (Equation 1b). Significant relationships are indicated by a regression line with gray CI bands. All graphs have the same *x* and *y* ranges. Trait values are kept in their standardized form (scaled and centered) to promote comparison between traits. Slopes and *p* values are presented in Table 2. Variables: RGR_sap95_, 95th percentile of sapling relative growth rates; LMA, leaf mass per area; WD, wood density; LAI_95_, 95th percentile of sapling leaf area index.

LMA and LAI_95_ had positive and rather large effects on RGR_sap95_. Increasing LMA or LAI_95_ by one SD increased RGR_sap95_ by 0.009 cm cm^−1^ year^−1^, which is 29% of the overall range of RGR_sap95_ in our study (0.044–0.075 cm cm^−1^ year^−1^; Table 2, model 1b). Also, in our alternative model, WD showed a negative but nonsignificant relationship with RGR_sap95_, and WD was not a significant predictor of RGR_sap95_ when used on its own (Table 3). Due to the weak effect of WD on growth, our alternative model did not always have a lower AIC_c_ score when compared to single‐trait models, as AIC_c_ penalizes extra variables unless they have a significant effect on growth (Table 3). This direction of the LMA–growth relationship and the absence of a WD–growth relationship are notably different from that reported in the prior literature (Figure 1).

(1c) $$\mathrm{RG}R_{\mathrm{sap}95,i}=\gamma_{0}+\gamma_{1}\mathrm{LM}A_{i}+\gamma_{2}\mathrm{LA}I_{95,i}+\epsilon_{3,i}.$$

**TABLE 3:** Results of single‐trait‐weighted least squares models of species potential growth rates (RGR_sap95_).

Model	*p*	*R* ^2^	AIC_c_	RMSE_w_	Intercept	Estimate	95% CI
LMA	0.15	0.21	−62.4	0.0059	0.051	0.0045	(−0.0020, 0.011)
WD	1.0	3.6 × 10^−8^	−59.7	0.0076	0.055	2.1 × 10^−6^	(−0.0083, 0.0083)
LAI_95_	0.37	0.090	−60.7	0.0067	0.059	0.0033	(−0.0046, 0.011)

*Note*: All predictor variables were standardized before analysis, while the response variable (growth rates, RGR_sap95_, in centimeters per centimeter per year) was kept in its original units. Coefficient estimates are in units of RGR_sap95_ which ranged between 0.044 and 0.075 cm cm^−1^ year^−1^.

Abbreviations: AIC_c_, Akaike information criterion for small sample sizes; LAI_95_ (in square centimeters per square centimeter), 95th percentile of sapling leaf area index; LMA (in kilograms per square meter), leaf mass per area; *p*, *p* value of the regression model; *R* ^2^, coefficient of determination; RMSE_w_, weighted root mean square error from leave‐one‐out cross‐validation analysis; WD (in grams per cubic centimeter), wood density;

**FIGURE 2:**
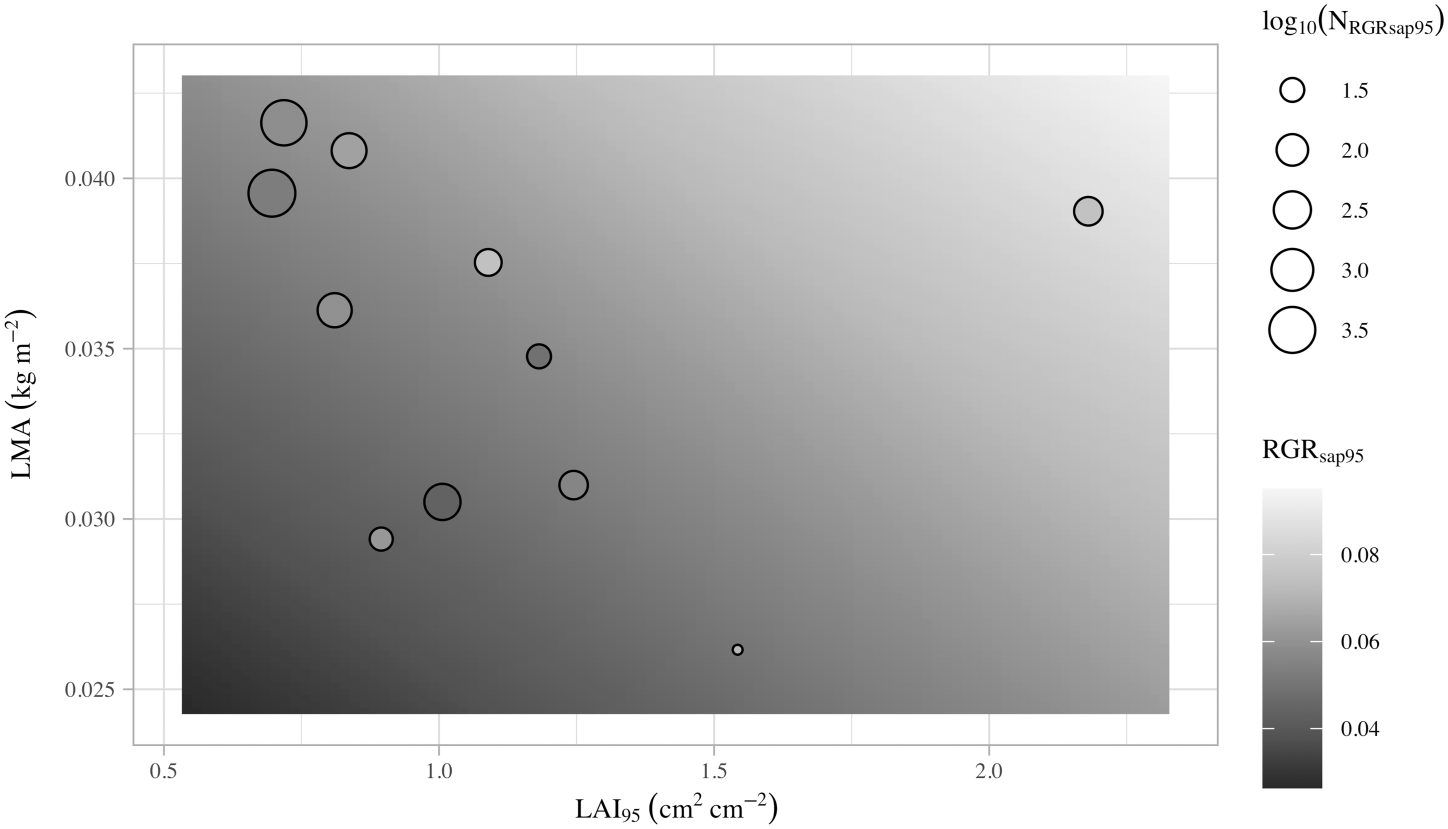
Negative correlation between species' leaf allocation (leaf area index, LAI_95_) and leaf morphology (leaf mass per area, LMA) that weakens the positive relationship of each on its own with RGR_sap95_ (weighted *r* = −0.56). The background gradient indicates the predicted RGR_sap95_ value (in centimeters per centimeter per year) of model 1c, highlighting the positive relationship between species' potential growth rate and each trait individually. Species' observed trait and growth values are indicated by markers. Marker size is determined by the number of observations used to calculate RGR_sap95_ (N_RGRsap95_) and marker shading is determined by species' RGR_sap95_. Due to the large range in sample sizes, we plotted marker size as the log_10_‐transformed sample size values. Deviations in color between markers and background gradient indicate differences between observed and predicted RGR_sap95_. The species with high LAI_95_ and LMA is *Quercus alba*.

Because WD was not a significant predictor of growth, we considered a version of the alternative model without it (Equation 1c, Figure 2). We found no significant differences between Equations (1b) and (1c) (*F*‐test *p* = 0.94), indicating that we could omit WD from our alternative model. This smaller alternative model (Equation 1c, *p* = 0.011, *R* ^2^_adj_ = 0.59, $F_{\text{stat}}=\frac{0.053}{0.0064}$ with 2 and 8 df) had a higher adjusted *R* ^2^ and lower AIC_c_ and RMSE scores than our initial alternative model, with LAI_95_ and LMA having the same positive influences on growth (Table 2). Partial *R* ^2^ of Equation (1c) showed that LMA and LAI_95_ explained 40% and 27% of the variation individually, respectively (unadjusted *R* ^2^ = 0.67).

Importantly, LMA and LAI_95_ alone were not significant predictors of RGR_sap95_ (Table 3). Additional *F*‐tests found that model 1c was significantly better than models of LMA (*F*‐test *p* = 0.010) and LAI_95_ (*F*‐test *p* = 0.0054) alone. These additional results beyond the success of our alternative model over our null further support the inclusion of leaf allocation (LAI_95_) and leaf morphology (LMA) together to best predict species' growth rates. These improvements in model performance were also supported by OLS regression (Appendix S1: Tables S3 and S4).

To understand why neither LMA nor LAI_95_ alone explained species' variability in RGR_sap95_, but a combined model has good performance with both predictors having strong effects, we examined the correlational structure between our traits (Appendix S1: Figure S6). Both LMA and LAI_95_ had a positive effect on growth rates but were negatively correlated with each other (weighted *r* = −0.56; *p* = 0.072; Figure 2). While this correlation was not strong enough to result in problematic multicollinearity according to VIF (Table 2), it was sufficiently strong to weaken the effects of either trait on growth rates unless both were included in the model. This can be understood by considering that species with higher LMA will have higher growth rates, but will also have lower LAI_95_ which would lower their growth rates, and vice versa. Including both LMA and LAI_95_ in the model eliminates these artifact effects through the other trait, allowing one to see their individual direct effects. Our best model (model 1c) predicted that the highest growth rate will be achieved when species have both high LMA and LAI_95_, but we rarely see that combination because most species fall along a negative LMA–LAI_95_ trade‐off (Figure 2).

## DISCUSSION

Functional traits have the potential to deepen our biological understanding of species' life history strategies. But there remain unexplored dimensions of traits' functionality, such as the role of leaf allocation, and less is known about trait–performance relationships in temperate forests. We asked to what extent incorporating both leaf allocation and leaf morphology improves our understanding of how leaf traits influence species' growth differences (Yang et al., 2018). We found that integrating leaf allocation into the model increased the amount of variance explained by about 50%, and this effect was driven by a negative correlation between leaf morphology (LMA) and leaf allocation (LAI_95_). Hence, considering these two traits simultaneously provides a more holistic understanding of plant functioning. We also found a positive LMA–growth relationship and lack of a relationship between growth and WD that contrasts with trends commonly found in tropical forests. In this temperate broadleaf winter‐deciduous forest, fast‐growing species had denser crowns (higher LAI_95_) and more conservative leaves (higher LMA). Below we discuss these results in more detail.

### Incorporation of leaf allocation with leaf morphology improves predictability of growth rates

Species' growth differences were best described by both LMA and LAI_95_ together (Equation 1c), which increased the amount of variation explained by about 50% (*R* ^2^_adj_ = 0.59) and improved the model (AIC_c_ = −66.8, RMSE_w_ = 0.0042). Within this model, LMA explained 40% of species' variation in growth (partial *R* ^2^), higher than values generally seen in the literature, which hover around 0.10 (Visser et al., 2016). These results support our hypothesis that beyond leaf morphology, how much plants allocate to photosynthetic tissue significantly improves estimates of species' growth rates. These patterns may be explained in part by the underlying negative correlation found between LMA and LAI_95_ (Figure 2). Low LMA leaves are cheaper to build and may allow species to build denser crown systems, suggesting a trade‐off in biomass allocation between leaf quantity and morphology. This permits species to achieve faster growth rates through different avenues, either by having many poor leaves (high LAI, low LMA) or by having fewer good leaves (low LAI, high LMA; Figure 2; Bonser, 2006). Therefore, a combination of leaf traits is needed to better model species' growth rates. Using only one dimension of leaf traits would bias estimates due to correlation with other traits and leave behind unexplained variations in growth.

Few studies have incorporated measures of leaf morphology together with leaf allocation to describe plant growth, but those that have agreed with our results. Li et al. (2017) found that the best regression model of plant growth contained CPA (a component of LAI) and specific leaf area (SLA, the inverse of LMA). Rubio et al. (2021) found significant trends when estimating growth using the product of LMA and CPA across all size classes. However, in our study, neither CPA + LMA (*p* = 0.21) nor CPA × LMA (*p* = 0.096) was a good predictor of sapling growth rates. In contrast with Li et al. (2017) and Rubio et al. (2021), our results with LAI_95_ suggest that including some measure of leaf quantity in our measure of leaf allocation, rather than crown size alone (CPA), may be necessary. Umaña et al. (2021) also found positive relationships between leaf area ratio (LAR) and leaf mass fraction (LMF) with growth rates in seedlings when incorporated with SLA, indicating that LAR and LMF may also be good metrics of leaf allocation. LAR and LMF are quantified as leaf area and leaf dry mass per whole plant dried biomass, respectively. As with LAI, LAR and LMF are measures of leaf quantity, though they require destructive sampling. In this study, we find that LAI_95_ together with LMA played an important role in modeling species' growth differences.

### Relationships between traits and growth rates

Our results also support the hypothesis that denser crowns promote faster growth rates among species at the sapling stage. LAI_95_ had a significantly positive influence on species' potential growth rates (Figure 1). This positive influence of the crown agrees with results found in some studies (Iida et al., 2014; Umaña et al., 2021), but disagrees with others (Li et al., 2017; Rubio et al., 2021). All of these studies were of tropical tree species, and we found almost no studies focused on broad‐leaved tree species in temperate deciduous forests. The exception is Takahashi et al. (2001), who found similar results to ours. They showed that mid‐successional species had larger crowns (higher CPA) and faster height growth than late‐successional species.

In these temperate forests, winter‐deciduousness results in annual leaf loss for all species (Runkle, 1989). We hypothesize that having denser crowns helps species to maximize their carbon assimilation and growth within each growing season before leaf fall. Prior work in Southern Michigan on the phenology of seedlings' carbon assimilation supports this idea. Lee and Ibáñez (2021) found that *Quercus rubra* seedlings had higher annual carbon assimilation than *Acer saccharum* because they maintained higher assimilation and respiratory rates during summer, while *A. saccharum* significantly reduced assimilation and respiration rates after spring. In our study, congeners *Q. alba* and *A. rubrum* had the highest and one of the lowest LAI_95_ values, respectively. This suggests that species with denser crowns at the seedling/sapling stage (e.g., *Quercus*) have higher respiratory costs and maintain high assimilation rates throughout the growing season, but can accumulate more carbon throughout the year, leading to faster growth rates. Species with sparser crowns (e.g., *Acer*) have lower respiratory rates and maintain low assimilation rates during summer under a closed canopy. They rely on reserves from high assimilation rates during spring to get them through the year, but ultimately have lower annual carbon assimilation and growth rates. Givnish (2020) also proposed that the multilayered crowns of early‐successional species in temperate forests reduce water loss in stomatal conductance and photoinhibition from self‐shading, reduce branching costs, and allow plants to take advantage of sidelighting, all of which promote faster growth rates. As all of our studied species are winter‐deciduous, differences in crown densities, branching costs, and assimilation strategies during the growing season should lead to different growth rates.

We found an unexpected trend with LMA, which positively correlated with species' growth rates. In tropical studies, prior work overwhelmingly supports a negative interspecific LMA–growth relationship (Medeiros et al., 2019; Wright et al., 2010). There, fast‐growing species often have broad, thin leaves that increase light interception and are cheap to build; slow‐growing species have thicker, better defended leaves that can be kept for multiple seasons. Among the few studies in temperate forests, we found additional evidence of this opposing trend in assemblages of temperate deciduous broadleaf tree species: Janse‐Ten Klooster et al. (2007) found that saplings with higher LMA had faster growth rates in Dutch forests, as did Fan et al. (2022) in China.

We hypothesize that greater desiccation risks and winter deciduousness in broadleaf temperate forests may generate LMA–growth relationships that are opposite to those found in tropical forests. Fast‐growing species found growing in more open and hotter microhabitats may invest in thicker leaves (high LMA) to reduce the risk of desiccation (Brodribb et al., 2007; Niinemets, 2001). Slow‐growing species overtopped by fast‐growing species may face less desiccation risk when growing in more shaded conditions. In that context, thinner, more acquisitive leaves (low LMA) may help saplings maximize carbon gain within a limited growing season at lower costs (Janse‐Ten Klooster et al., 2007), allowing them to allocate their carbon elsewhere instead of investing heavily in leaves that are annually dropped. However, our study did not quantify light and humidity conditions and cannot test these ideas.

We found an insignificant but negative relationship between WD and growth (Figure 1b). Prior studies in species‐rich communities often find a significantly negative WD–growth relationship (Chave et al., 2009; Visser et al., 2016; Wright et al., 2010). While it is possible that the mismatch in scale between our measurements of WD (continental scale) and growth (site‐specific) may have weakened the WD–growth relationships, we note that WD generally exhibits low variability within species (Siefert et al., 2015), and prior work comparing local WD measurements with literature values found similar values across between scales (Muller‐Landau, 2004). We also considered whether ontogenetic stage may have influenced this relationship, with the hypothesis that leaf traits may play a greater role than wood traits at juvenile stages. We re‐ran our analyses for adult trees (dbh≥10cm; Appendix S1: Section S4), and found that WD was also a nonsignificant predictor of dbh growth rates at the adult stage.

We propose two potential explanations for the weak WD–growth relationship in this study. First, WD is weakly connected to plant hydraulics, and therefore may not be closely linked to plant functions related to growth that are under selective pressures (Fan et al., 2012; Russo et al., 2010). In this forest, drought stress during summers and freezing during winters may select for hydraulic characteristics that are more important for growth and disconnected from WD. Second, allometric relationships suggest that height increases more rapidly than diameter at smaller diameter sizes (Feldpausch et al., 2011). Saplings may prioritize vertical growth to compete for light, leading to greater variation in height growth compared to diameter growth. WD does negatively correlate with interspecific variation in juvenile height growth in both tropical and temperate studies (Janse‐Ten Klooster et al., 2007; Visser et al., 2016). We lack height data to examine these trends in this study, but propose that height growth is important to consider in future studies.

Finally, we note that our study site is a secondary forest undergoing mesophication. The trait and demographic strategy structure of forests are both known to vary with successional stage (Poorter et al., 2019; Umaña et al., 2023) and disturbance frequency (Russo et al., 2020), and hence both of these characteristics of the forest may also contribute to the realization of trait–performance relationships different from those often found in the literature.

### Statistical limitations in species‐poor communities

Studying species‐poor communities reduces the statistical power we have to detect trait–performance relationships relative to species‐rich communities, such as those in tropical forests. However, while our sample size is limited, our analyses describe the overall characteristics of this temperate forest because our studied species make up the majority of the community (86.6% of living stems). Many of the excluded species were rare and belonged to two genera already present in our analyses (*Quercus* and *Fraxinus*), and their inclusion would not add much phylogenetic diversity to our study. Furthermore, our study provides important functional trait data for the oldest temperate forest in the ForestGEO network (https://forestgeo.si.edu/). Evidence of contrasting trends in leaf–performance relationships presented here suggests that results from tropical studies cannot be generalized to temperate forests, and that further studies in temperate forests are necessary. We suggest that analyses that aggregate species across multiple forests could be a fruitful avenue for future research of temperate communities.

### Trait–demography relationships, species richness, and coexistence

These interspecific differences in functionally important traits may promote stable coexistence in communities (Chesson, 2000). Differences in leaf allocation, leaf morphology, and wood traits are important for describing plant species' partitioning of gradients in light and other limiting resources (Iida et al., 2014; Wright et al., 2010), and drive species' variation along life history strategy trade‐offs (Adler et al., 2013). Such niche partitioning is thought to be an important mechanism of stable coexistence in plant communities (Kohyama, 1993; Pacala & Rees, 1998). Additionally, potential trade‐offs between traits themselves (e.g., LAI_95_ and LMA) can generate a diversity of trait combinations that may have an equalizing effect on species fitness and further increase species richness in communities (Marks & Lechowicz, 2006). By strengthening our understanding of relationships between traits and demographic rates, we are able to generalize and understand trends across species, facilitating ecological generalizations that promote finding general rules in community ecology (McGill et al., 2006).

## AUTHOR CONTRIBUTIONS

Minh Chau N. Ho conceptualized the study. Minh Chau N. Ho and María Natalia Umaña designed the study and collected the data, with help from many research assistants acknowledged below. Minh Chau N. Ho revised and modified the code to calculate growth rates from the E. S. George Reserve census data. Minh Chau N. Ho, Michael Kalyuzhny, and Annette M. Ostling analyzed the data and interpreted results. Minh Chau N. Ho drafted the manuscript, and all authors contributed to revisions.

## CONFLICT OF INTEREST STATEMENT

The authors declare no conflicts of interest.

## Supporting information


Appendix S1:

## Data Availability

Data for leaf morphology, crown allocation, and species' maximum growth rates (Ho et al., 2025) are available in the University of Michigan's Deep Blue Data archive at https://doi.org/10.7302/s7hm-h824. Raw census data for species' growth rates (Allen et al., 2019) are available in the University of Michigan's Deep Blue Data archive at https://doi.org/10.7302/WX55-KT18. Wood density data (Zanne et al., 2009) are available in Dryad at https://doi.org/10.5061/DRYAD.234.
