# Supplementary material for: Leaf allocation improves predictability of interspecific growth rates in a broadleaf deciduous temperate forest
Source: Ecology. 2025 Sep 13;106(9):e70203. doi: 10.1002/ecy.70203 (PMC12432972; doi:10.1002/ecy.70203)
Supplement: Supplementary file 1 — Appendix S1: [file ECY-106-e70203-s001.pdf]

Appendix S1

Journal name: Ecology

Manuscript title: Leaf allocation improves predictability of interspecific growth rates in a broadleaf deciduous temperate forest

Authors:

Minh Chau N. Ho, Michael Kalyuzhny, María Natalia Umaña, Annette M. Ostling

Section S1. Species-specific DBH_{sap} cut-off

Table S1. DBH cut-off that determines the max size threshold for saplings (DBH_{sap}, cm) for the studied deciduous temperate tree species in the Michigan Big Woods (Michigan, U.S.), based on literature values whenever possible or inferred from congeners. References: a) Minor and Kobe (2017), b) Sakai (1990), c) Nielsen and Kjær (2010), d) Carr and Banas (2000), e) Suzuki *et al.* (2019), f) Schlesinger (1990).

Family	Latin Name	Authority	Common Name	DBH_{sap}
Rosaceae	*Prunus serotina*	Ehrhart	black cherry	7.5[a]
Sapindaceae	*Acer rubrum*	Linnaeus	red maple	5.0[b]
Rosaceae	*Amelanchier arborea*	(F. Michx.) Fernald	serviceberry	3.5
Fagaceae	*Quercus alba*	Linnaeus	white oak	7.5[a]
Ulmaceae	*Ulmus americana*	Linnaeus	American elm	5.0[c]
Lauraceae	*Sassafras albidum*	(Nuttall) Nees	sassafras	5.0
Cornaceae	*Cornus florida*	Linnaeus	flowering dogwood	3.5[d]
Betulaceae	*Ostrya virginiana*	(Miller) K. Koch	hophornbeam	7.5[e]
Tiliaceae	*Tilia americana*	Linnaeus	American basswood	7.5[a]
Fagaceae	*Fagus grandifolia*	Ehrhart	American beech	7.5[a]
Oleaceae	*Fraxinus americana*	Linnaeus	white ash	7.5[f]

For each species, we defined saplings as individuals with initial $DBH_i \leq DBH_{sap}$, a species-specific DBH cut-off informed by the literature (Table S1). In general, understory species had a cutoff of 3.5cm DBH_i, mid-sized tree species at 5.0cm, and all other taller species at 7.5cm. For many species, DBH_i is below the size at which fruits and flowers were first noticed amongst individuals surveyed in Michigan (Minor and Kobe 2017; Sakai 1990); some species values were estimated in Virginia (*Cornus florida*; Carr and Banas 2000) or North America more generally (*Fraxinus americana;* Schlesinger 1990). For *Ulmus americana* and *Ostrya virginiana*, we were unable to find species-specific reproductive size, and referenced our cut-off values from congeners in Denmark and Japan, respectively (Nielsen and Kjær 2010; Suzuki *et al.* 2019). For *Amelanchier arborea*, an understory tree, we used the same 3.5cm cut-off used for *C. florida*, another understory species (Barnes and Wagner 2004). For *Sassafras albidum*, a medium-sized

tree from a species-poor genus (Griggs 1990), we were unable to find species-specific or congener values and opted for an intermediate 5cm cut-off.

Section S2. Species-level differences in LAI_{95}

Species' LAI_{95} values ranged from 0.70 cm^2 cm^{-2} (*Prunus serotina*) to 2.18 cm^2 cm^{-2} (*Quercus alba*) (Appendix S2: Table S1). *P. serotina* (black cherry) and *Acer rubrum* (red maple), which had small LAI_{95} and slower growth rates, had small leaves and wide crowns (large CPA). *Q. alba* (white oak) and *Fraxinus americana* (white ash), with high LAI_{95} and faster growth rates, had large leaves and relatively smaller crowns. Compared to leaf mass per area (LMA), intraspecific LAI distributions had longer upper tails, and species seem to vary more in their upper tails than in their means (Appendix S2: Figure S1). While species may overlap in their average LAI, they differed in their maximum potential crown densities as quantified by LAI_{95}, which makes LAI_{95} a more useful metric of species characteristics.

Note that intraspecific CV is higher for LAI than LMA because LAI data was collected throughout the plot (*i.e.*, across a heterogenous abiotic environment) while LMA was collected from leaves in high light environments only. For LAI, the intraspecific variation is not directly comparable to interspecific variation, as our interspecific variation was quantified from LAI_{95}.

Table S2. Trait and growth values for each species, including mean, intraspecific, and interspecific coefficients of variation (CV, the ratio of standard deviation to the mean times 100%). For LAI_{95} and RGR_{sap95}, we present the 95th percentile of each species. Interspecific CV of species' LAI and RGR values were calculated across LAI_{95} and RGR_{sap95}, respectively, and across mean species' values for all other traits. Wood density data is from Zanne *et al.* (2009) and does not include intraspecific CV.

	LAI		LMA		WD			
**Latin Name**	**LAI_{95}**	**CV**	**Mean**	**CV**	**Mean**	**CV**	**RGR_{sap95}**	**RGR_{tre95}**
Acer rubrum	0.72	45.95	0.042	11.90	0.49		0.059	0.057
Amelanchier arborea	0.81	45.29	0.036	23.91	0.66		0.059	0.032
Cornus florida	1.18	34.66	0.035	7.03	0.64		0.049	0.014
Fagus grandifolia	1.09	45.68	0.038	33.50	0.56		0.075	0.062
Fraxinus americana	1.54	38.95	0.026	13.38	0.55		0.071	NA
Ostrya virginiana	1.01	40.68	0.031	16.80	0.63		0.044	0.034
Prunus serotina	0.70	38.87	0.040	10.68	0.47		0.053	0.036
Quercus alba	2.18	43.17	0.039	8.81	0.60		0.075	0.018
Sassafras albidum	1.24	36.97	0.031	13.79	0.42		0.056	0.044
Tilia americana	0.89	33.88	0.029	15.47	0.32		0.062	0.063
Ulmus americana	0.84	39.65	0.041	13.09	0.46		0.065	0.052
**Interspecific CV**	39.33		14.73		20.02		16.74	41.89

Variables: LAI ($cm^2\ cm^{-2}$), sapling leaf area index; LAI_{95} ($cm^2\ cm^{-2}$), 95th percentile of LAI; LMA ($kg\ m^{-2}$), leaf mass per area; WD ($g\ cm^{-3}$), wood density; RGR_{sap95} ($cm\ cm^{-1}\ yr^{-1}$), 95th percentile of saplings' relative growth rate; RGR_{tre95} ($cm\ cm^{-1}\ yr^{-1}$), 95th percentile of adults' relative growth rate.

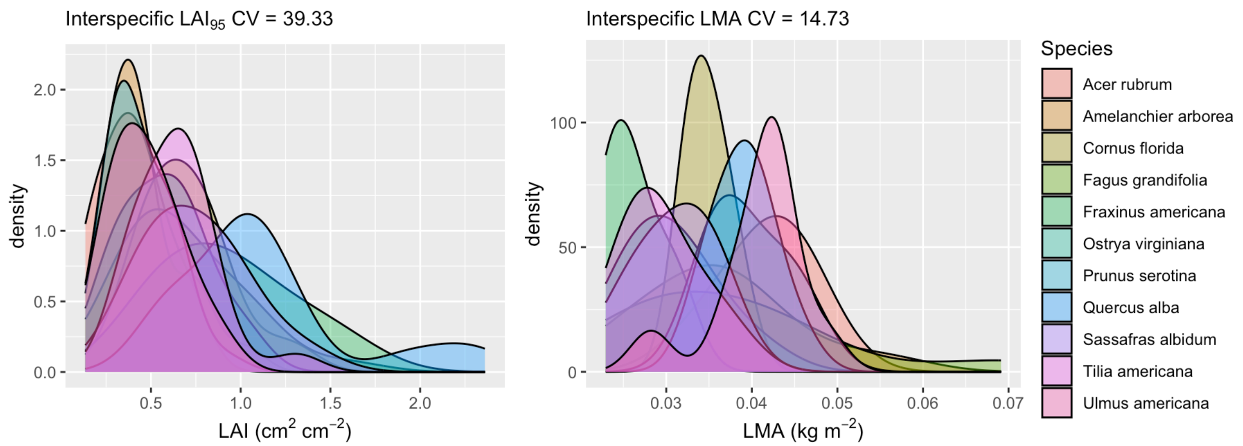

Figure S1. Density plots of leaf and crown traits, for each species. For leaf mass per area (LMA), coefficient of variation (CV) across species (using species' means) are presented here. For LAI, interspecific CV was calculated using species' LAI_{95}. For both leaf traits, intraspecific CV is provided in Appendix S2: Table S1. Note that LAI data was collected throughout the plot (*i.e.*, across a heterogenous abiotic environment) while other variables were collected from leaves in high light environments only. *Variables*: LAI ($cm^2\ cm^{-2}$), sapling leaf area index; LAI_{95} ($cm^2\ cm^{-2}$), 95th percentile of LAI; LMA ($kg\ m^{-2}$), leaf mass per area.

Section S3. Ordinary least squares regression

In the main text, all regression models were ran using weighted least squares to account for differences in species' abundance when calculating species' maximum potential growth rates (RGR_{sap95}). Here, we present the same models using ordinary least squares (OLS). None of our models were significant models of interspecific growth variations (Appendix S3: Tables S1, S2). Adding LAI_{95} did not improve models' predictions of RGR_{sap95} (F-test, P=0.069; Appendix S3: Table S2), although this result was marginally non-significant and AICc scores suggest that models including LAI_{95} was a better fit than models without. As in WLS models, wood density was not a significant predictor and could be removed; model 1b was not significantly better than model 1c (F-test, P=0.32), and had a larger AICc score.

Table S3. Results of single-trait models of species potential growth rates (RGR_{sap95}) using ordinary least squares (OLS) models. All predictor variables were standardized before analysis, while the response variable (growth rates, RGR_{sap95}, cm cm^{-1} yr^{-1}) was kept in its original units. Coefficient estimates are in units of RGR_{sap95} which ranged between 0.044-0.075 cm cm^{-1} yr^{-1}.

Model	P	R^2	AICc	RMSE	Intercept	Estimate	95% CI
LMA	0.70	0.018	-61.6	0.01203	0.061	0.0013	(-0.0062, 0.0089)
WD	0.80	0.0076	-61.5	0.01131	0.061	-0.00088	(-0.0085, 0.0067)
LAI_{95}	0.14	0.22	-64.2	0.009827	0.061	0.0048	(-0.0019, 0.012)

Abbreviations: P – p-value of the regression model; R^2 – coefficient of determination; AICc, Akaike information criterion for small sample sizes; RMSE, root mean square error from leave-one-out cross-validation analysis; CI, confidence interval. *Variables*: LAI_{95} (cm^2 cm^{-2}), 95th percentile of sapling leaf area index; LMA (kg m^{-2}), leaf mass per area; WD (g cm^{-3}), wood density.

Table S4. Ordinary least squares (OLS) models of species' potential growth rates (RGR_{sap95}). Weighted least squares (WLS) model results are presented in the main text. All predictor variables were standardized before analysis, while the response variable (growth rates, RGR_{sap95}, cm cm^{-1} yr^{-1}) was kept in its original units. Coefficient estimates are partial regression coefficient estimate of each variable, in units of RGR_{sap95} which ranged between 0.044-0.075 cm cm^{-1} yr^{-1}.

									Predictor estimates (95% CI)		
Model		***P***	**R^2_{adj}**	**F-test**	**AICc**	**RMSE**	**VIF**	**Int.**	**LMA**	**WD**	**LAI_{95}**
1a	LMA + WD	0.89	-0.22		-56.5	0.01285	1.01	0.061	0.0014 (*P*=0.70) (-0.0067, 0.0096)	-0.0010 (*P*=0.78) (-0.0092, 0.0072)	
1b	LMA + WD + LAI_{95}	0.26	0.16	0.069	-54.7	0.01091	1.18-1.28	0.061	0.0041 (*P*=0.24) (-0.0035, 0.012)	-0.0033 (*P*=0.32) (-0.011, 0.0041)	0.0071 (*P*=0.069) (-0.00071, 0.015)
1c	LMA + LAI95	0.21	0.15	0.32	-60.4	0.01026	1.13	0.061	0.0033 (*P*=0.32) (-0.0039, 0.011)		0.0059 (*P*=0.096) (-0.0013, 0.013)

Abbreviations: *P*, p-values; R^2_{adj}, adjusted coefficient of determination; F-test, model 1a versus 1b, and model 1b versus 1c, p-values; AICc, Akaike information criterion for small sample sizes; RMSE, root mean square error from leave-one-out cross-validation analysis; VIF, variance inflation factor of covariates, values ≥ 5 are considered problematic; Int., model intercept; CI, confidence interval. *Variables*: LMA (kg m^{-2}), leaf mass per area; WD (g cm^{-3}), wood density; LAI_{95} (cm^2 cm^{-2}), 95th percentile of sapling leaf area index.

Section S4. Ontogeny and adult growth rates

In our main text, we focused our analyses on the relationship between traits and sapling potential growth rates (RGR_{sap95}). At this ontogenetic stage, wood density (WD) was not a significant predictor of sapling potential growth rates.

Here we consider how our leaf and wood traits correlate with species' adult potential growth rates (RGR_{tre95}), calculated as the 95th percentile of diameter relative growth rates ($RGR_{tre} = \ln\left(\frac{DBH_f}{DBH_i}\right)/(t_f - t_i)$). For every species, we identified adult individuals as those with diameter at breast height $DBH \geq 10cm$, and we pooled RGR_{tre} measurements across the 2003-2008 and 2008-2014 census intervals. One species, *Fraxinus americana*, had only one RGR_{tre} observation and was excluded from our analyses; the remaining ten species had 9-2949 RGR_{tre} observations with an average of 666.8. We ran two analyses. First, we examined the correlation between RGR_{tre95} and RGR_{sap95} to understand how relative growth rates may have changed with ontogeny. Second, we examined the relationships between RGR_{tre95} and leaf mass per area (LMA), leaf area index (LAI_{95}), and WD using the same null and alternative hypotheses framework presented in our main results. Regression models were ran using weighted least squares, all predictor variables were standardized before analysis, and the response variable (RGR_{tre95}) was kept in its original units, which ranged between 0.014-0.062 cm cm^{-1} yr^{-1}.

Adult and sapling RGR_{95} appeared to be positively correlated (Figure S2), but the irregular growth pattern of *Quercus alba* skewed the correlation to be non-significant (r=0.29, *P*=0.41). In general, all species had slower potential growth rates as adults, though *Q. alba* had much slower adult potential growth rate relative to its juvenile potential growth rates.

Overall, traits (LMA, LAI_{95}, and WD) were poor predictors of RGR_{tre95} (Table S5). None of our models were good descriptors of the growth data (model *P*=0.10-0.21; single-trait model

results not shown). This weakening of growth-trait relationships with increasing tree size has been found in prior studies (Wright *et al.* 2010; Visser *et al.* 2016). At adult stages, other factors (*e.g.*, allocation to reproduction) may reduce the significance of allocation to leaves and wood for growth. We find a similar trend in our study, where LMA and LAI_{95} were strong predictors of sapling potential growth rates but were not significant predictors of adult potential growth rates. Wood density remains a non-significant predictor of growth rates at both ontogenetic stages examined in this forest.

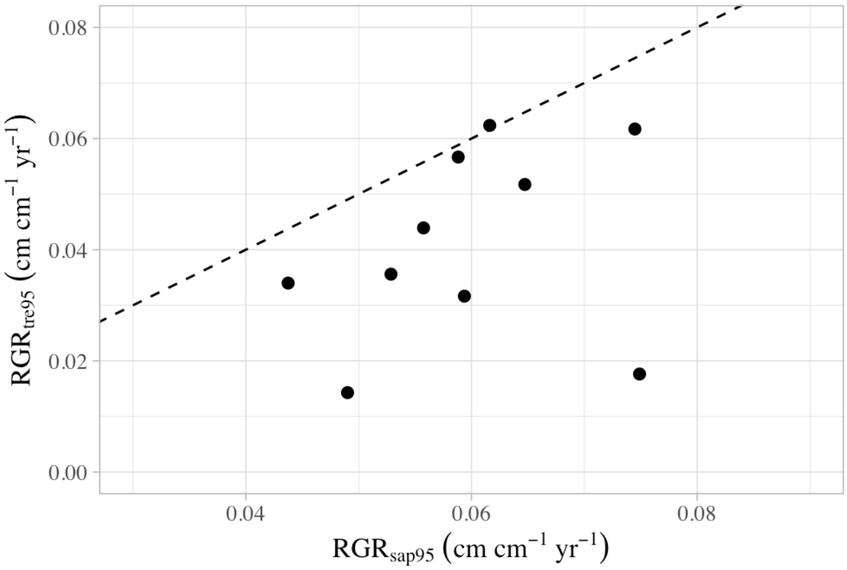

Figure S2. Relationships between adult potential growth rates (RGR_{tre95}, cm cm^{-1} yr^{-1}) and sapling potential growth rates (RGR_{sap95}, cm cm^{-1} yr^{-1}). A 1:1 line is represented as a dashed line.

Table S5. Weighted least squares models of species' adult potential growth rates (RGR_{tre95}). All predictor variables were standardized before analysis, while the response variable (growth rates, RGR_{tre95}, cm cm^{-1} yr^{-1}) was kept in its original units. Coefficient estimates are partial regression coefficient estimate of each variable, in units of RGR_{tre95} which ranged between 0.014-0.062 cm cm^{-1} yr^{-1}.

									Predictor estimates (95% CI)		
Model		***P***	**R^2_{adj}**	**F-test**	**AICc**	**$RMSE_w$**	**VIF**	**Int.**	**LMA**	**WD**	**LAI_{95}**
2a	LMA + WD	0.12	0.31		-33.0	0.022	1.0053	0.028	0.0077 (*P*=0.32) (-0.0095, 0.025)	-0.016 (*P*=0.057) (-0.032, 0.00063)	
2b	LMA + WD + LAI_{95}	0.21	0.26	0.49	-24.9	0.036	1.57-3.83	0.032	0.0036 (*P*=0.72) (-0.019, 0.027)	-0.0075 (*P*=0.59) (-0.040, 0.025)	-0.0044 (*P*=0.49) (-0.019, 0.010)
2c	LMA + LAI_{95}	0.10	0.33	0.59	-33.4	0.034	1.12	0.035	0.00072 (*P*=0.93) (-0.017, 0.019)		-0.0072 (***P*=0.050**) (-0.014, -0.000017)

Abbreviation: *P*, p-values, with bolded values significant at $\alpha = 0.05$; R^2_{adj}, adjusted coefficient of determination; F-test, model 2a versus 2b, and model 2b versus 2c, p-values; AICc, Akaike information criterion for small sample sizes; $RMSE_w$, weighted root mean square error from leave-one-out cross-validation analysis; VIF, variance inflation factor of covariates, values ≥ 5 are considered problematic; Int., model intercept; CI, confidence interval. *Variables*: LMA (kg m^{-2}), leaf mass per area; WD (g cm^{-3}), wood density; LAI_{95} (cm^2 cm^{-2}), 95th percentile of sapling leaf area index.

Section S5: Supplementary figures

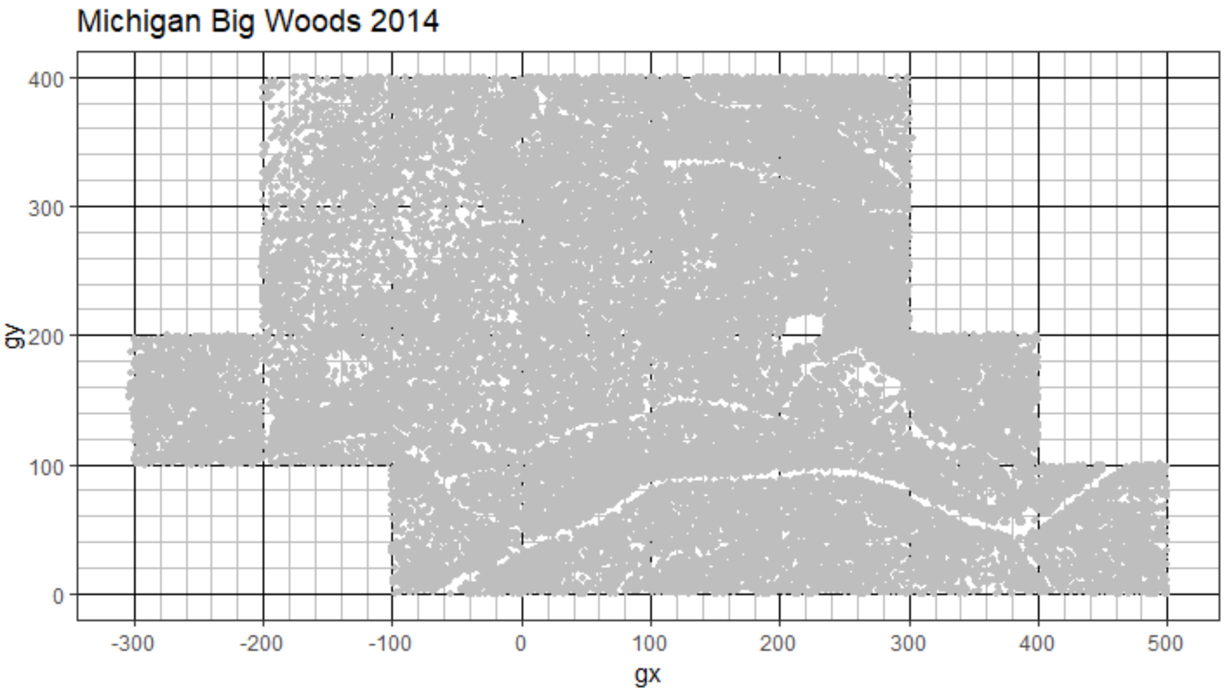

Figure S3. All stems in the plot in 2014, with roads visible in as empty lines occurring sparsely throughout the plot.

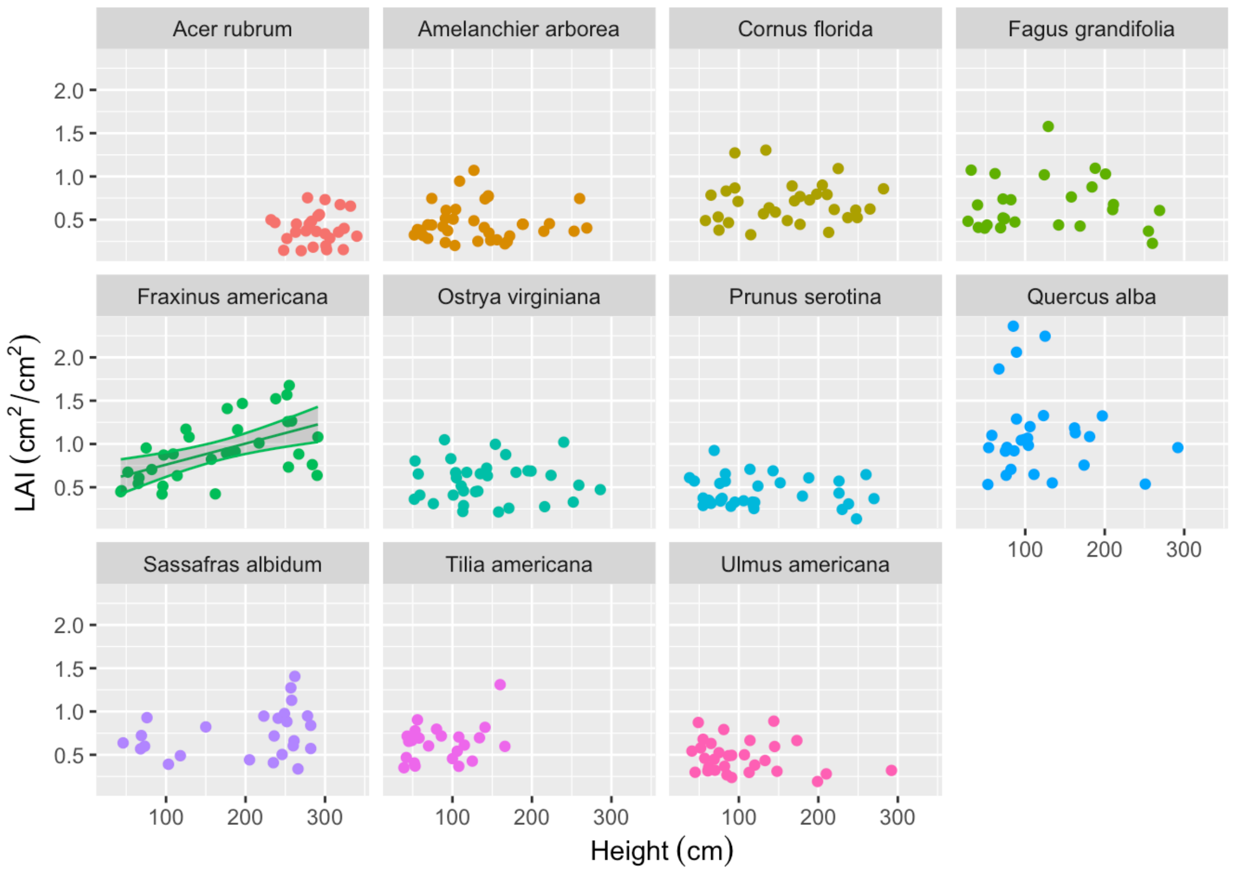

Figure S4. Allometric relationship between LAI and plant height. For most species, there was not a significant relationship between plant size (as proxied by height) and LAI. Only *Fraxinus americana* showed a significant relationship (P = 0.0010, R^2 = 0.31).

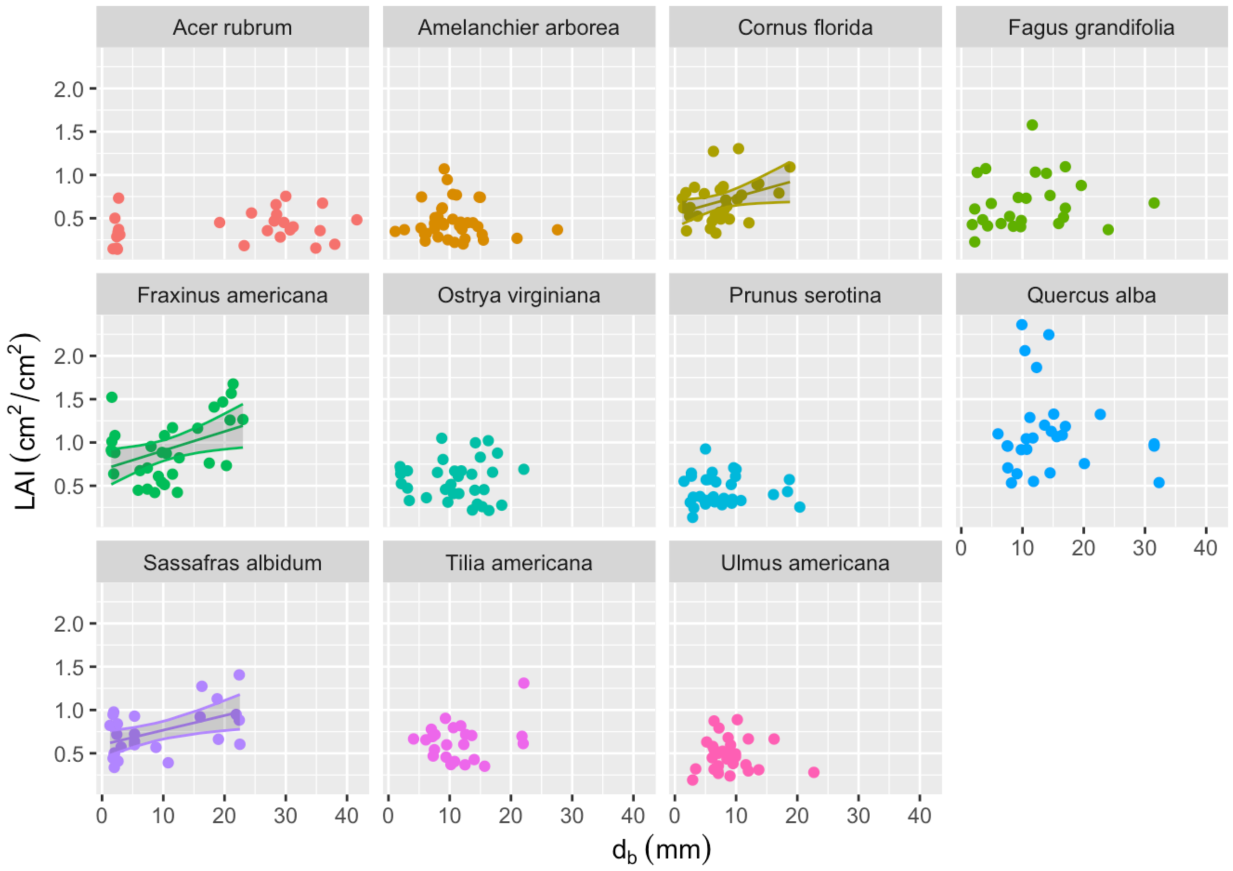

Figure S5. Allometric relationship between leaf area index (LAI) and stem basal diameter (d_b). Most measured saplings were not large enough to have a meaningful DBH, so we opted to represent stem diameter using d_b. For most species, there was not a significant relationship between plant size (as proxied by d_b) and LAI, with the exception of *Cornus florida* (P = 0.41, R^2 = 0.14), *Fraxinus americana* (P = 0.018, R^2 = 0.17), and *Sassafras albidum* (P = 0.012, R^2 = 0.25).

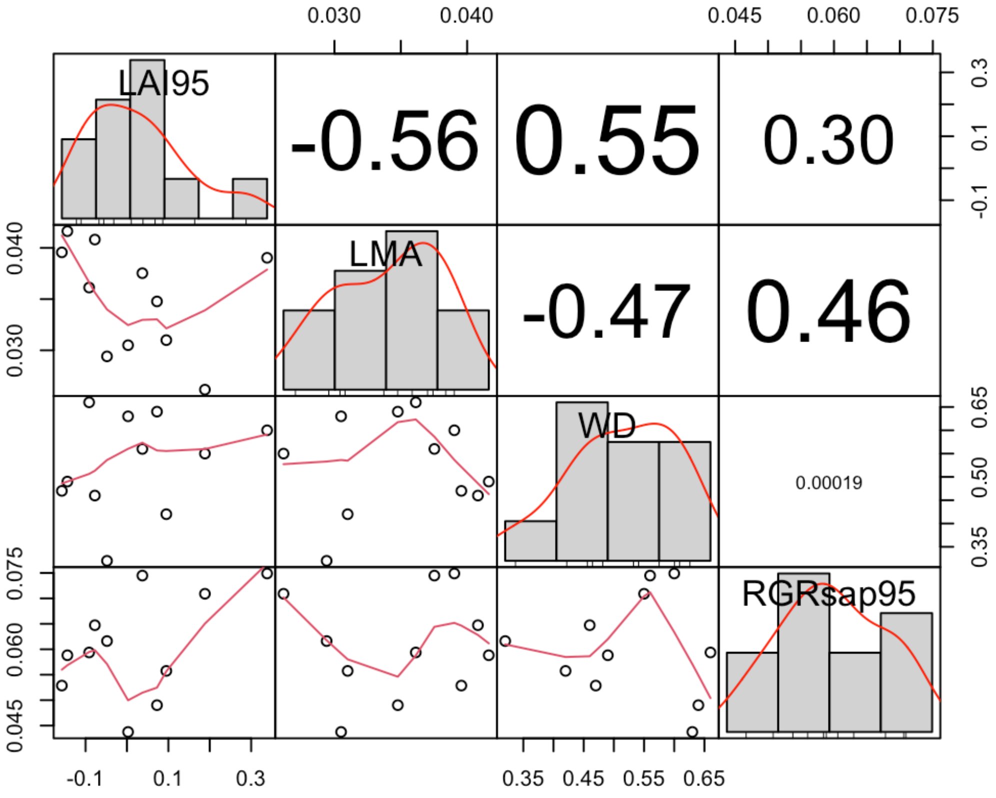

Figure S6. Pearson's weighted correlations between traits. Variables: LAI_{95} (cm^2 cm^{-2}), 95th percentile of sapling leaf area index, log_{10}-transformed; LMA (kg m^{-2}), leaf mass per area; WD (g cm^{-3}), wood density.

References

Barnes, Burton V., and Warren H. Wagner. 2004. *Michigan Trees: A Guide to the Trees of the Great Lakes Region*. Ann Arbor, MI: The University of Michigan Press. https://press.umich.edu/Books/M/Michigan-Trees-Revised-and-Updated.

Carr, David E., and Lauren E. Banas. 2000. "Dogwood Anthracnose (Discula Destructiva): Effects of and Consequences for Host (Cornus Florida) Demography." *The American Midland Naturalist* 143 (1): 169–77. https://doi.org/10.1674/0003-0031(2000)143[0169:DADDEO]2.0.CO;2.

Griggs, Margene M. 1990. "Sassafras Albidum (Nutt.) Nees." In *Silvics of North America*, edited by Russell M. Burns and Barbara H. Honkala, 2. Hardwoods:773–77. Agriculture Handbook 654. United States Department of Agriculture, Forest Service. https://www.srs.fs.usda.gov/pubs/misc/ag_654_vol2.pdf.

Minor, David M., and Richard K. Kobe. 2017. "Masting Synchrony in Northern Hardwood Forests: Super-Producers Govern Population Fruit Production." *Journal of Ecology* 105 (4): 987–98. https://doi.org/10.1111/1365-2745.12729.

Nielsen, Lene Rostgaard, and Erik Dahl Kjær. 2010. "Gene Flow and Mating Patterns in Individuals of Wych Elm (Ulmus Glabra) in Forest and Open Land after the Influence of Dutch Elm Disease." *Conservation Genetics* 11 (1): 257–68. https://doi.org/10.1007/s10592-009-0028-5.

Sakai, Ann K. 1990. "Sex Ratios of Red Maple (Acer Rubrum) Populations in Northern Lower Michigan." *Ecology* 71 (2): 571–80. https://doi.org/10.2307/1940310.

Schlesinger, Richard C. 1990. "Fraxinus Americana L. White Ash." In *Silvics of North America*, edited by Russell M. Burns and Barbara H. Honkala, 2. Hardwoods:333–38. Agriculture

Handbook 654. United States Department of Agriculture, Forest Service. https://www.srs.fs.usda.gov/pubs/misc/ag_654_vol2.pdf.

Suzuki, Maki, Kiyoshi Umeki, Olga Orman, Mitsue Shibata, Hiroshi Tanaka, Shigeo Iida, Tohru Nakashizuka, and Takashi Masaki. 2019. “When and Why Do Trees Begin to Decrease Their Resource Allocation to Apical Growth? The Importance of the Reproductive Onset.” *Oecologia* 191 (1): 39–49. https://doi.org/10.1007/s00442-019-04477-y.

Visser, Marco D., Marjolein Bruijning, S. Joseph Wright, Helene C. Muller-Landau, Eelke Jongejans, Liza S. Comita, and Hans de Kroon. 2016. “Functional Traits as Predictors of Vital Rates across the Life Cycle of Tropical Trees.” *Functional Ecology* 30 (2): 168–80. https://doi.org/10.1111/1365-2435.12621.

Wright, S. Joseph, Kaoru Kitajima, Nathan J. B. Kraft, Peter B. Reich, Ian J. Wright, Daniel E. Bunker, Richard Condit, et al. 2010. “Functional Traits and the Growth–Mortality Trade-off in Tropical Trees.” *Ecology* 91 (12): 3664–74. https://doi.org/10.1890/09-2335.1.

Zanne, Amy E., G. Lopez-Gonzalez, David A. Coomes, Jugo Ilic, Steven Jansen, Simon L. Lewis, Regis B. Miller, Nathan G. Swenson, Michael C. Wiemann, and Jerome Chave. 2009. “Data from: Towards a Worldwide Wood Economics Spectrum.” Dryad. https://doi.org/10.5061/DRYAD.234.
